# Development of tartaric esters as bifunctional additives of methanol-gasoline

**DOI:** 10.1186/1752-153X-8-25

**Published:** 2014-04-15

**Authors:** Jie Zhang, Changchun Yang, Ying Tang, Rui Zhou, Xiaoli Wang, Lianghong Xu

**Affiliations:** 1 College of Chemistry and Chemical Engineering, Xi’an Shiyou University, Xi’an, Shaanxi 710065, People’s Republic of China; 2 College of Petroleum Engineering, China University of Petroleum-Beijing, Beijing 102249, People’s Republic of China; 3 Shaanxi Yanchang Zhongli New Energy Co. Ltd, Xi’an, Shaanxi 710065, People’s Republic of China

**Keywords:** Methanol-gasoline, Tartaric esters, Phase stability, Evaporation

## Abstract

**Background:**

Methanol has become an alternative fuel for gasoline, which is facing a rapidly rising world demand with a limited oil supply. Methanol-gasoline has been used in China, but phase stability and vapor lock still need to be resolved in methanol-gasoline applications. In this paper, a series of tartaric esters were synthesized and used as phase stabilizers and saturation vapor pressure depressors for methanol-gasoline.

**Results:**

The results showed that the phase stabilities of tartaric esters for methanol-gasoline depend on the length of the alkoxy group. Several tartaric esters were found to be effective in various gasoline-methanol blends, and the tartaric esters display high capacity to depress the saturation vapor pressure of methanol-gasoline.

**Conclusion:**

According to the results, it can be concluded that the tartaric esters have great potential to be bifunctional gasoline-methanol additives.

## Background

Rapidly rising world demand, together with the limited oil supply, means developing clean and alternative fuels increasingly draws worldwide attention [1]. Among the alternative fuels, methanol displays fine combustion properties similar to gasoline and has such advantages as high octane number, low emissions, antiknock, rich resource, and mature technology, so it can be used as an alternative fuel for gasoline [2]. In recent years, extensive research of the low percentage methanol-gasoline has been carried out, and it has been applied in Shanxi, Sichuan, Zhejiang, Inner Mongolia, Shaanxi, Xinjiang and other provinces of China gradually [3]. However, there are several problems needing to resolve in methanol-gasoline application, in which the phase stability is the first and most important one. One of the popular solutions is to add phase stabilizer to reduce alcohol-oil interfacial tension [4,5], such as ethers, ketones, esters, fatty alcohols, aliphatic hydrocarbons, fatty acids, non-ionic surfactants, acetal/ketones, biodiesels and amidines [6-8]. Secondly, the low boil point of methanol leads to high possibility of vapor lock by raising the vapor pressure of methanol-gasoline [9-11]. The current solution for vapor lock is to add pressure depressor, such as aliphatic ketones, lynn classes, fatty aldehydes, fatty ethers, acetals/ketals, etc. At present, few researches have researched bifunctional additives with the functions of improving the phase stability as well as depressing the effective vapor pressure of the methanol-gasoline mixtures. In this work, a series of tartaric esters was synthesized and screened in the methanol-gasoline as a bifunctional additive for the phase stability and vapor depressor.

## Finding

Tartaric esters were synthesized by two methods with high yield. The purified tartaric esters were evaluated as phase stabilizer and saturation vapor pressure depressor of methanol-gasoline. The results show that the efficiency depends on the length of the tartaric esters’ alkoxy group. With the dosage of 0.1%, all tartaric esters can depress the saturation vapor pressure lower than that of gasoline, and decyl tartaric is the most effective one.

## Experimental

### Materials and methods

All solvents were AR grade and purchased from Xi’an Chemical Agent Co, and the 93^#^ gasoline is commercially available. The phase stabilizing and pressure reducing tests were carried out on DFY-cryostat instrument (Xi'an Yuhui Instrument Co.Ltd.) and DSL-080 vapor pressure detector (Dalian the Ceon Electronic Equipment Co.Ltd.).

### Synthesis of tartaric esters

The short-chain alcohols can co-dissolved with water, so the produced water can not be separated from the reactant, while long-chain alcohol can carry the produced water out under reflux. So different methods were used for the synthesis of tartaric esters.

Method A [12]: Tartaric acid (23 g), methanol (18 mL), and *p*-toluenesulfonic acid (TsOH) (0.6 g) were added in a 250 ml flask. Refluxing for 10 h, the mixture was cooled to room temperature. Methanol and methyl tartaric were distillated respectively. The synthesis of ethyl tartaric and propyl tartaric is similar to method above.

Method B [13,14]: Tartaric acid (0.15 mol), *n*-butanol (0.45 mol), cyclohexane (30 mL), *p*-toluenesulfonic acid (0.5 g) were added in a 250 ml flask equipped with a water separator. Refluxing for 5 h, the mixture was cooled to room temperature, cyclohexane, *n*-butanol and *n*-butyl tartaric were separated by vacuum distillation. The synthesis of amyl tartaric, hexyl tartaric, hepyl tartaric, octyl tartaric, decyl tartaric is similar to method above.

### Phase stability test

The fuel blends were prepared by blending 15, 30, 50 and 65 vol. % of methanol with base gasoline, and the gasoline blends were assigned as M15, M30, M50 and M65. The phase stabilizing tests were carried out according to Chinese National standards of GB 8017–87, GB/T 23799–2009, DB61/T 352–2004 and DB51/T 448–2004. First the test tube full of methanol-gasoline with different ratios was placed in a cryostat, and then the temperature was adjusted from 40°C to −25°C. At each degree, the tube was taken out and was shaken for two to three seconds, and the phase separation temperature was determined as the solution becomes cloudy [15,16]. The tests were repeated until the separation temperature does not change for 3 times.

### Vapor pressure test

Effect of tartaric esters on vapor pressure of methanol-gasoline was investigated according to Chinese standards of GB 8017–87. The methanol-gasoline was poured into the vapor pressure detector and put into the water bath of 37.8°C. The methanol-gasoline was intensive mixed by taking the detector from the water bath every 5 min and reversing violently. The operation was repeated until the pressure does not change for 3 times.

## Results and discussion

### Synthesis of tartaric esters

**Scheme 1:**
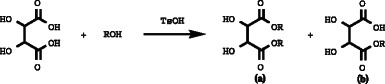
The reaction of tartaric acid and alcohol The reaction of tartaric acid and alcohol.

The reactions of tartaric acid and alcohols are shown in Scheme 1, and both the reaction conditions and the yield are summarized in Table 1. In this reaction, diester (a) is the main product, and the monoester (b) is the undesired byproduct. To reduce the byproduct, high quantities of alcohol are used. For the synthesis of the first three esters, the alcohols were employed with high ratio over 30: 1 to tartaric acid. For the rest esters, less alcohol is needed with the 5: 1 molar ratio of alcohol to tartaric acid, because the alcohols with more than 4 carbon atoms can compose azeotrope with water which can be separated by a water separator, so less alcohol is needed. To get purified tartaric esters for the following experiments phase stability and vapor pressure test, the synthesized esters were purified by vacuum distillation. The yields were obtained in the range from 60.2% to 77.6% as shown in Table 1[17-19].

**Table 1:** The results of the synthesis of tartaric esters

**Esters**	**Tartaric acid : alcohol**	**Method**	**Yield (%)**
Methyl tartaric	1: 30	A	70.9
Ethyl tartaric	1: 30	A	71.1
Propyl tartaric	1: 30	A	76.5
Butyl tartaric	1: 5	B	77.6
Amyl tartaric	1: 5	B	64.5
Hexyl tartaric	1: 5	B	60.2
Hepyl tartaric	1: 5	B	72.1
Octyl tartaric	1: 5	B	71.5
Decyl tartaric	1: 5	B	72.2

### Effect of tartaric ester on the phase stability of methanol-gasoline

**Figure 1:**
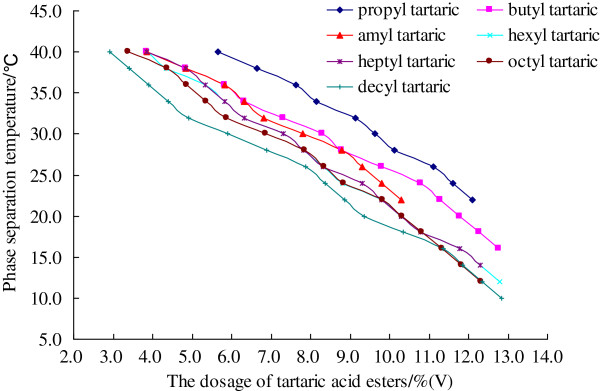
The effect of the tartaric ester dosage on the phase stability of M15.

**Figure 2:**
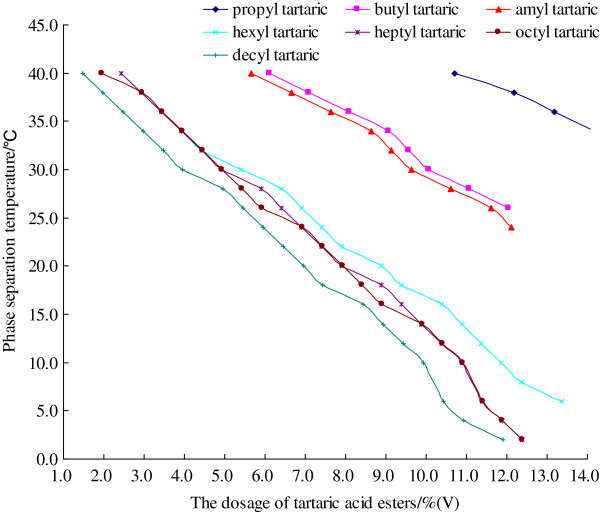
The effect of the tartaric ester dosage on the phase stability of M30.

**Figure 3:**
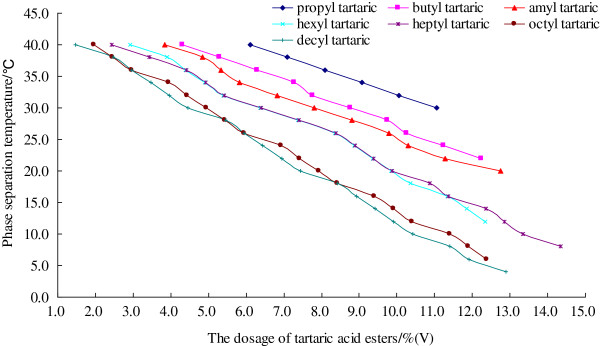
The effect of the tartaric ester dosage on the phase stability of M50.

**Figure 4:**
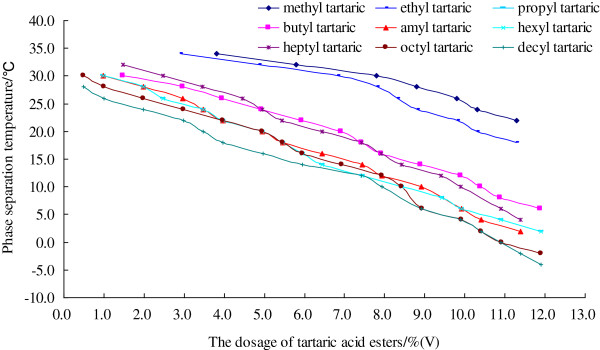
The effect of the tartaric ester dosage on the phase stability of M65.

**Figure 5:**
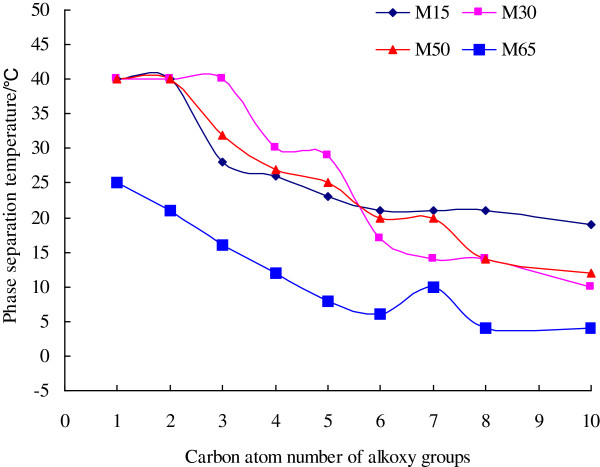
The relationship of the alkoxy groups and the phase separation temperature.

The phase stabilities of tartaric esters for the methanol-gasoline blends of M15, M30, M50 and M65 at different temperatures from −25°C to 40°C were investigated and summarized in Figures 1, 2, 3 and 4. The results indicate that the length of alkoxy group of tartaric ester effects on the phase stability for methanol-gasoline significantly. For the esters with short alkoxy group, such as methyl tartaric and ethyl tartaric, the phase stability for methanol-gasoline are ineffective, even as the dosage over 10%, methanol and gasoline does not homogenize to produce M15, M30, M50 and M65 at 40°C. The reason may be due to the strong hydrophilicity but weak lipophilicity of short-carbon-chained tartaric ester, leading them hard to dissolve in gasoline. By increasing the carbon chain of tartaric esters, lipophilicity of the esters is markedly enhanced, and the dissolvent in gasoline is intensified, resulting in higher solubility for the various blends. According to the results, it can be found that long-carbon-chained tartaric esters are effective in phase stability to methanol-gasoline. The phase separation temperatures of the four methanol-gasoline blends with the ester dosage of 10% were estimated and shown in Figure 5. It can be found that the phase separation temperature declines along with the length of the alkoxy group. For M15, the lowest phase separation temperature was obtained as hexyl tartaric ester was employed. For M30, the optimal carbon atom number of alkoxy group is 7. For M50, the optimal carbon atom number of alkoxy group is 8. For M65, the lowest phase separation temperature was obtained as the carbon atom number of alkoxy group is 8.

### The effect of tartaric ester on the evaporation of methanol-gasoline

**Figure 6:**
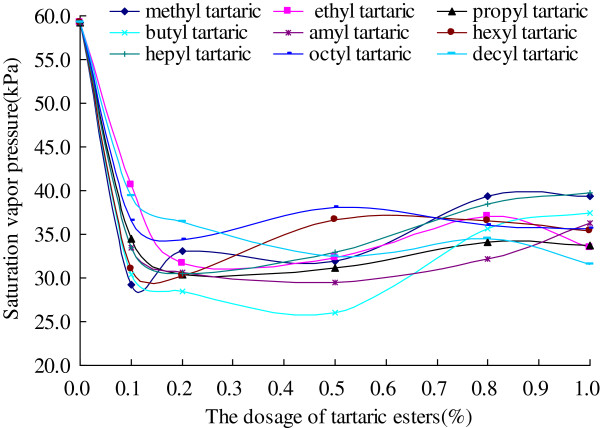
The effect of tartaric esters on the evaporation of methanol-gasoline M15 system.

**Figure 7:**
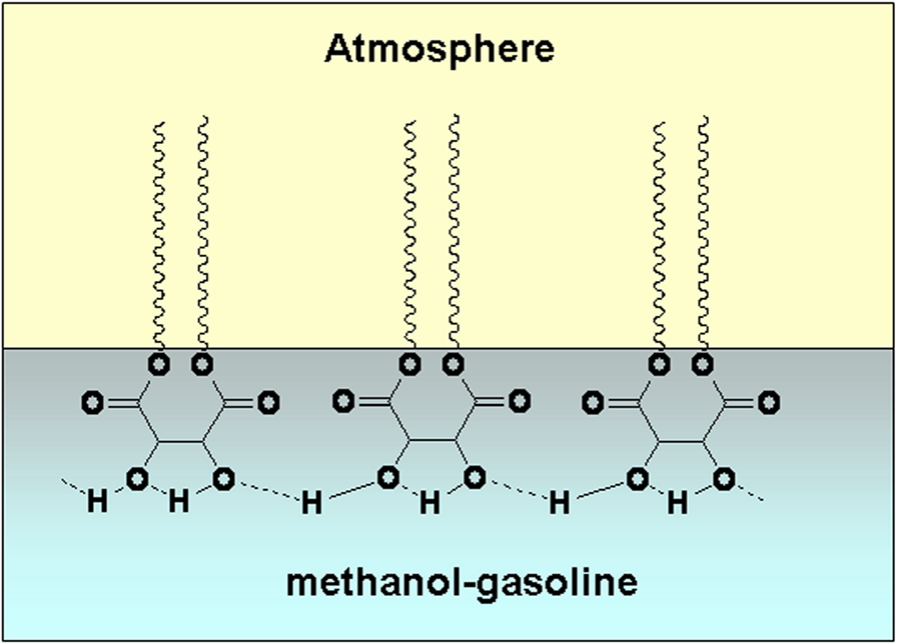
Distribution of ester molecules on the interface of methanol-gasoline and atmosphere.

The saturation vapor pressure will rise extremely over gasoline as it blends with low percentage methanol such as M15 and M30, which will lead to vapor block as it used under relative high temperature. Some chemicals with lower saturation vapor pressure have been employed to depress the high pressure of gasoline. In this work, the effect of tartaric ester on the saturation vapor pressure of M15 methanol-gasoline was investigated referred to GB 8017–87 “petroleum products the vapor pressure determination method (Reid Method)”, and the results are shown in Figure 6. The original saturation vapor pressure of M15 is 63.5 kPa, which is 5.7 kPa higher than that of gasoline. As little amount of esters were added in, the saturation vapor pressure was depressed obviously. With the esters’ dosage of 0.1%, methyl tartaric, ethyl tartaric, propyl tartaric, butyl tartaric, amyl tartaric, hexyl tartaric and decyl tartaric can depress the saturation vapor pressure lower than that of gasoline, among which decyl tartaric is the most effective one. Further increase of the dosage depresses the saturation vapor pressure ineffectively. The main reason may contribute to the distribution of tartaric esters on the surface of methanol-gasoline as shown in Figure 7. The tartaric esters alkoxy groups point to the hydrophobic part, gas phase, and the hydroxyl groups and the carboxyl groups point to the less hydrophobic part, methanol-gasoline, linking each other by hydroxyl bounds, which can prevent the formation of an azeotrope with low boiling point.

## Conclusions

Tartaric esters were synthesized and screened for their performances of phase stabilizing in M15, M30, M50 and M65 and pressure reducing in M15. The results show that the length of alkoxy group of tartaric esters effects on the phase stability of methanol-gasoline significantly. The phase stability of tartaric esters for the methanol-gasoline system with long length is more potent than that with short length. All of the synthesized esters are potent to depress the saturation vapor pressure of methanol-gasoline. With the dosage of 0.1%, all tartaric esters can depress the saturation vapor pressure lower than that of gasoline, and decyl tartaric is the most effective one.

## Competing interests

The authors declare that they have no competing interests.

## Authors’ contributions

Jie Zhang carried out the synthesis of tartaric esters, and Changchun Yang participated in using phase stabilizer and saturation vapor pressure depressor of methanol-gasoline. Ying Tang, Rui Zhou, Lianghong Xu and Xiaoli Wang evaluated that the stabilities of the methanol-gasoline depend on the length of the tartaric esters’alkoxy group. All authors have read and approved the final manuscript.
